# Lyophilization: a useful approach to the automation of analytical processes?

**DOI:** 10.1155/S1463924690000347

**Published:** 1990

**Authors:** M. D. Luque de Castro, A. Izquierdo

**Affiliations:** Department of Analytical Chemistry Faculty of Sciences University of Córdoba Córdoba 14004 Spain

## Abstract

An overview of the state-of-the-art in the use of lyophilization for the pretreatment of samples and standards prior to their storage and/or preconcentration is presented. The different analytical applications of this process are dealt with according to the type of material (reagent, standard, samples) and matrix involved.


Journal of Automatic Chemistry, Vol. 12, No. 6 (November–December 1990), pp. 267–279

### Lyophilization: a useful approach to the automation of analytical processes?

**M. D. Luque de Castro and A. Izquierdo**
*Department of Analytical Chemistry, Faculty of Sciences, University of Córdoba, 14004 Córdoba, Spain*

*An overview of the state-of-the-art in the use of lyophilization for the pretreatment of samples and standards prior to their storage and/or preconcentration is presented. The different analytical applications of this process are dealt with according to the type of material (reagent, standard, samples) and matrix involved.*

### Introduction

Analytical instrumentation is growing steadily in complexity and degree of automation [1–4]. Such automation normally affects the later steps of the analytical process (analytical reaction, signal measurement and transducing, and data acquisition and processing), rather than the preliminary operations (sampling and sample treatment), which pose special difficulties on account of the large variety of samples that can be encountered in each state of aggregation (solid, liquid, gas) and in different particle sizes; the diversity of conditions (location of the spot and distance to the laboratory, storage requirements); and the type of pretreatment required (dissolution, preconcentration, interference removal). All these often make the first stage of the analytical process difficult to automate; in fact, endeavours in this field are frequently aimed at a specific type of sample or application (for example clinical, food, agricultural or pharmaceutical analysis).

At present, work on automatic methods of analysis is focused on these preliminary steps, which involve performing partial or complete automatic dissolution by means of electrical energy [5, 6], ultrasound [7, 8] and other types of energy, and applying automatic continuous separation techniques [9, 10].

Lyophilization is an alternative to the automation of sample pretreatment. As the terms 'lyophilization' and 'freezing' are frequently used in this paper, both are defined below for clarification. Lyophilization is an operation by which water (or another component) is separated by sublimation from a frozen system or phase. The passage from solid to gas occurs without the appearance of water or the solvent in liquid state. Freezing is the process by which water becomes congealed into ice by cold.

In this paper freeze-drying processes for analytical purposes are reviewed. The contradictory results obtained by different authors mean that no final conclusions can be drawn. Nevertheless, this paper could be a starting point for viewing this technique as a means of automating sample pretreatment by designing general or specific approaches to solving some of the problems posed by the automation of preliminary steps.

This review has been divided into different parts according to whether freeze-drying is used for storage and/or preconcentration of reagents, standards or analytes (whether inorganic or organic). The nature of the sample matrix exerts marked influences on the behaviour of the analytes; thus, a subclassification taking into account the nature of the (biological [clinical, agricultural, food] or aqueous) has also been made. Special emphasis is placed on drug analysis. Finally, different instruments for lyophilization, and earlier reviews, are discussed.

### Reagents

The storage of reagents poses peculiar problems, particularly in clinical analysis. A major difficulty in evaluating the results of immunochemical analyses is the lack of a readily available reference reagent [11]. This shortcoming seems to have been partly solved by the use of lyophilized reagents. The large number of patents applied for in this field in the past decade testify to the potential of these processes in immunochemical analysis. Reported examples in this context include the use of filter paper strips on polystyrene film impregnated with buffer; β-galactosidase and theophylline antisera that were then lyophilized for the determination of theophylline [12]; the lyophilization of mixtures of incompatible reagents in the presence of water, such as those required for the haemagglutination test of hCG (chorionic gonadotropin) in pregnancy [13]; and the use of a series of reagents frequently employed in immunoassays (for example, peroxidase [14], β-microglobulin-phosphatase-labelled antibody [15, 16], anti-IgG (immunoglobulin G) antibody anti-insulin antibody complex [17], and sensitized thyroglobulin [18], among others). A series of lyophilized reagent kits for detection of human T-cell leukaemia virus specific antibodies [19], immunochemical assay for chorionic gonadotropin [20], for production of lyophilized plasma membrane-receptor preparation [21], and for the determination of toxicants and antibodies [22] have been patented.

The water in lyophilized radiopharmaceutical kits can be determined by using a straightforward Karl Fischer apparatus [23].

A method for the determination of ferritin based on sandwich immunoassay and the use of lyophilized reagents was successfully applied and then patented [24]. On the other hand, the use of lyophilized membranes for radioreceptor assay of opiates and opiod peptides is also

0142–0453/90 $3.00 © 1990 Taylor & Francis Ltd.

of great interest [25], as are other generic reagents [26] and tracers[27].

An interesting study by McCarthy *et al.* [28] showed the major improvement resulting from lyophilization with such reference reagents as IgG aggregates. Previous studies performed by Kauffman *et al.* [29] on the stability of this reagent showed that, although size fractioned heat-aggregated IgG could be successfully used as a reference reagent, the material which they tested was frozen at −70 °C and was unstable in the absence of 0·5% (w/v) bovine serum albumin. To overcome these shortcomings, IgG aggregates produced by McCarthy *et al.*, by heating gamma globulin solutions, were freeze-dried, kept at 4 °C and reconstituted up to 4 months later. Compared with frozen (−20 °C) preparations, only minimal changes in biological reactivity and in physical integrity were found to occur during this period. These results showed the potential use of freeze-dried preparations of heat-aggregated IgG as reference reagents for the comparative evaluation and standardization of immune complex assays.

Technicon patented a method for lyophilizing reagent-coated particles, which preserved both the reagent activity and the particle suspension dispersity by using a combined suspension of the particles and a zwitterionic buffer as stabilizer, and a cryoprotective agent followed by the combined suspension. The results obtained were excellent [30].

The microbial production of $NAD^+$ (nicotinamide adenine dinucleotide) for clinical and biochemical analysis was accomplished by incubating nicotinic acid and/or adenosine, AMP (adenosine monophosphate), ADP (adenosine diphosphate), and ATP (adenosine triphosphate) with freeze-dried NAD-producing microorganisms. The results obtained prompted the authors to patent their system [31]. Lyophilic analytical reagents based on starch have also been obtained with this type of process [32].

### Standards

The most extensive use of freeze-drying for the preparation and storage of standards is in the clinical field, particularly for serum standards and controls to be used with different types of analytes.

Control serum used in the quality control of clinical chemistry must be stable over a comparatively long period of time to be useful and meaningful. Only lyophilization or freezing at temperatures close to −30 °C have thus far proved practical for this purpose. Both methods of preservation have been extensively used in clinical laboratories. Comparative studies of both methods have been performed. Formerly, lyophilization was considered to offer no advantages with regard to levels of glucose, urea, nitrogen, sodium, potassium, total protein and serum glutamic oxalacetic transaminase [33]. Although it is widely accepted that lyophilization will almost certainly alter the protein matrix of serum because of changes in tertiary structures, recent detailed studies contradict this assumption. Thus Clark *et al.* used isotachophoresis to study the effects of lyophilization on the protein matrix of quality-control sera [34], and concluded that the observed differences in the isocatophoresis traces of the quality-control sera may arise from a number of factors. First, the lyophilization process itself may account for some of these changes, which was supported by the results of this study. Second, interspecies variation in serum proteins were found to occur [35]. Third, treatment during the manufacturing process (for example dialysis and supplementing with chemicals and tissue extracts) may change protein matrices. Finally, the storage conditions, the presence of proteolytic bacteria and the 'ageing' of serum may result in changes in the protein constituents.

The preparation of control sera containing lipo- and apolipoproteins poses special problems. Plasma lipoproteins cannot be preserved and stored in liquid form for a very long time. Furthermore, some lipoproteins become insoluble after lyophilization; thus the reconstituted serum is turbid, which interferes with spectrophotometric measurements. Therefore, quality-control material is usually de-lipoproteinated, and, consequently, contains only low concentrations of cholesterol and triglycerides. Most lipoproteins are affected by lyophilization and may no longer correspond to native lipoproteins, which makes their assessment by electrophoretic or immunological methods difficult. Wieland and Seidel proposed a simple procedure by which serum lipoproteins are completely protected from denaturation during lyophilization [36]. The procedure involves adding sucrose to serum or a plasma control before lyophilizing (or freezing). The final concentration of sucrose should be between 413 and 825 mmol/l and can be added in solid form or dissolved. The mechanism by which sucrose protects lipoproteins from the denaturation induced by lyophilization is unclear. It is probably incorporated into the hydration shell of the molecule and replaces water during the freeze-drying process. On reconstitution, it may readily accept water, with the consequent restoration of the shell. The methods most markedly influenced by the physico-chemical state of the analyte of interest are lipoprotein electrophoresis and the rate-nephelometric determination of apolipoprotein B. With both of these methods, results with the lyophilized control serum cannot be distinguished from those of fresh whole serum. Preparation of lyophilized human serum based reference material with graded levels of apolypoproteins A-1 and B is of great interest on the account of the role of these compounds as indicators of the risk of coronary heart disease [36–39]. Henderson *et al.* [40] used proven techniques employing ethyl alcohol and acetate buffer to precipitate either apolipoprotein A-1 rich or apolipoprotein B rich fractions that were blended with whole or delipidated serum producing five pilot-sized pools containing graded levels of the apolipoproteins. After lyophilization, the pools were tested and all pools were found to contain analyte concentrations similar to frozen serum and variable amounts of apolipoproteins A-1 and B. Temporal and accelerated thermal stability testing demonstrated that the analytes in the pools could withstand the passage of time (3 years) and high temperatures (up to 56 °C). This technology thus lends itself for use as a preparative procedure for apolipoprotein reference materials over the extended range needed in clinical applications.

The usefulness of lyophilized human serum for quality control in the determination of lactate dehydrogenase isoenzymes and total activity was demonstrated by Degtyar *et al.* [41]; on the other hand, long-term stability studies of enzymes, total protein, and inorganic analytes in lyophilized quality control serum performed by Lawson *et al.* [42] showed total protein to be the most universally stable, the behaviour of the enzyme activity of the different enzymes varying over wide margins. Thus, while the enzyme activity of alkaline phosphatase tended to increase and that of creatine phosphokinase generally decreased, glutamic oxalacetic transaminase and lactate dehydrogenase were very erratic in their behaviour. The concentrations of inorganic species, such as chloride and potassium, increased minimally, and the changes in the calcium concentration were very inconsistent. As a rule, the ratios between the rate of change in the analyte value to the standard deviation were lower for electrolytes than for other analytes.

General studies on osmometric estimation of vial-to-vial variation in contents of lyophilized serum [43], the atmosphere within sealed glass ampules containing lyophilized biological material [44], storable universal control serum containing normal and pathological ranges of enzymes, substrates, and metabolites [45], methods to prepare whole blood control sample [46], and cautions in the use of methods for calibrating glucose with lyophilized material [47], demonstrate the interest in lyophilization in the clinical field.

Lyophilized standard reference materials for urine samples are as common as those for serum and blood samples. Recently, the National Institute of Standards and Technology (NIST) has prepared and certified SRM 1507, a freeze-dried urine fortified with $\Delta$11-nor-$\Delta^9$-tetrahydrocannabinol-9-carboxylic acid (THC-9-COOH), the major urinary metabolite of marijuana. The certified concentration of 20 $\pm$ 1 ng/ml for the analyte was obtained from the consistent results of analyses of the material by gas chromatography/mass spectrometry (GC/MS) and high-performance liquid chromatography with electrochemical detection (HPLC/EC). Solid-phase extraction was used to prepare the sample for GC/MS analyses, and liquid-liquid extraction was used for the HPLC/EC analyses. The multistep HPLC method was developed at the NIST to overcome interferences from urine constituents. The results of a 'round-robin' test on this material involving five departments of Defense Laboratories involved in drug testing were used for certification [48]. The possibility of producing fruit-juice reference materials is conditioned by the availability of a method allowing the original juice or concentrate to be transformed into a physically more stable form.

Many international standards, reference preparations and reference reagents are routinely prepared by lyophilization in the presence of inert carriers, followed by an extensive period of secondary desiccation. Bristow *et al.* [49] used high-performance liquid chromatography to analyse the effects of lyophilization and secondary desiccation on the initial degradation and subsequent stability of a model protein (insulin). Secondary desiccation was found to promote a reaction of insulin with a carrier consisting of non-volatile buffer salts and a sugar. However, secondary desiccation did not improve the stability of insulin according to accelerated thermal degradation and analysis using the Arrhenius equation. The authors concluded that careful consideration needs to be given, on a case-by-case basis, to the selection of the procedures for the preparation of international standards, particularly those ampouled in the absence of carrier proteins and intended for physicochemical analysis, such as HPLC [49]. Reference materials to meet multipurpose needs for analysis of both inorganic and organic constituents in biological investigations are not readily available. A human total diet material has been investigated as a possible reference material for a wide variety of constituents of interest in human nutrition and health. This material showed a stable assay value for the natural levels of a number of vitamins following freeze-drying or radiation sterilization. This is an important feature in producing reference materials, which will be stable over the long term, for natural levels of these constituents. An exception is an increase by 34% in the assay value of folic acid upon freeze-drying and an 85% increase upon freeze-drying followed by radiation sterilization [50]. The feasibility of transforming of orange juice by freeze-drying was studied. As a rule, the powder obtained is hygroscopic and tends to agglomerate into large solid lumps. However, under optimal conditions of freeze-drying, combined with redrying of the powder before closing the bottles, a slightly cohesive, but physically stable, yellow orange powder can be obtained. Moreover, most amino acids (asparagine, methionine, lysine and arginine) are not affected by freeze-drying. Based on the study of a limited number of important fruit juice parameters, we may conclude that freeze-drying seems to be a very promising technique for obtaining an orange juice reference material preserving the original chemical properties of orange juice after redissolution in water [51].

Recently, Versieck *et al.* [52] certified a second-generation biological reference material (freeze-dried human serum) for trace element determinations. The material was prepared under rigorously controlled conditions to avoid extraneous additions. Analytical data were obtained by the authors, as well as by numerous other intra- and extra-mural investigators, solicited on the basis of established experience in determining selected elements. For 14 trace elements (Al, Cr, Mn, Fe, Co, Cu, Zn, As, Se, Br, Rb, Mo, Cd and Cs) certified values (in ng/g or µg/g dry weight) were listed; for an additional element (Ni) a best estimate (in ng/g dry-weight) was added. Trace element concentrations in the material, which is available to the scientific community, closely approximated those in normaly lyophilized blood plasma or serum. The material thus provides a means of checking the accuracy and precision of analytical procedures for quantifying low-level trace elements in the best possible conditions, and for detecting errors that can readily be overlooked when reference materials with higher levels of trace elements are used. In addition, and in contrast to already existing biological reference materials with high levels of trace elements, it offers the possibility of identifying unsuspected errors at the sample preparation stage.

Standards for X-ray fluorescence analysis have been prepared in different ways depending on the analyte concerned. Thus, a method for preparing standard solutions for uranium was prepared by weighing to an accuracy of 0·1%, which corrects for variations in aliquoting and for evaporation loss during weighing. The standards were freeze-dried in a configuration suited to X-ray fluorescence analysis [53]. Membrane filter standards were prepared for Cu, Zn, Ni, Pb and Mn for calibration in X-ray fluorescence spectrometers [54].

### Samples

Lyophilization has also been used in sample pretreatment, particularly for sample preservation, but also for preconcentration. Its use depends on the type of analyte (organic or inorganic) to be assayed after restitution of the sample, which, in summary, determines the usefulness of the pretreatment step. Each group of analytes has been further divided according to the nature of the sample matrix (inorganic and organic, both from plants and animals).

### *Inorganic analytes*

Metal traces and subtraces have so far been the most frequently determined inorganic compounds in biological samples, both in solid [55–66] and in liquid [67–69] materials by radiochemical [55–57, 64–67], X-ray [61, 62, 69, 70] and atomic absorption spectrometric techniques [68].

### *Biological solid samples*

High specific activity radio-isotopes of chromium, zinc and selenium were used by Fourte and Peisach [55] to label these elements accumulated by the oyster *Crassostrea gigas*. The retention of the metabolized forms of these elements during freeze-drying or oven-drying at 50 °C, 90 °C, 105 °C and 120 °C was studied. Experimental results showed that accepted procedures for dehydration of zoological material, even at relatively low temperatures, may involve losses of trace elements. Previous assumptions that metabolized trace elements would behave analytically like inorganic materials were shown to be valid in some cases, but open to question in others. The work points to a serious lack of information on possible organo-metallic compounds that may be formed during the metabolism. Yield determinations with inorganic salts commonly used to establish the accuracy of a method may therefore be unacceptable in principle. Preliminary results in support of these conclusions have already been obtained for Mn, Fe, Co, Cd and Pb, but more comprehensive results will be published shortly.

Chromium(III) and Cr(VI) losses during freeze-drying and oven-drying at different temperatures from various rat tissues and faeces containing radioactive isotopes were assayed by Iyengar *et al.* [56]. Significant losses of Cr(III) occurred from fur samples (hair and skin) on freeze-drying. Oven-drying at 80 °C caused no losses of either form of Cr, but at 105 °C there were minor losses from kidney and faeces samples. At 120 °C, Cr(III) was lost from all samples to varying extents, whereas losses of Cr(VI) were less significant. The overall error made in drying the samples even at 120 °C did not exceed 10–15%, even for the most markedly affected tissues.

Xue and Wang [57] studied the loss of lead from various kinds of biological materials following different drying and ashing procedures by using $^{203}Pb$ as a tracer, which was intravenously injected into mice. No loss in any samples was observed after freezing or oven-drying. Ashing caused no loss of $^{203}Pb$ from the samples by either oxygen plasma or oven-drying, but the retention of lead on the beaker wall was quite different. Following the oxygen plasma ashing, the leaching rate of lead in 6M HCl could reach 100%. On the other hand, 50–70% of $^{203}Pb$ in serum, red cells and scalp hair remained on the beaker wall after oven-drying at 650 °C, but no retention of $^{203}Pb$ on the beaker wall was observed in the case of liver, kidney or lung. The recovery of $^{203}Pb$ approached 100% on using high-pressure digestion. On the other hand, Nakaguchi *et al.* found that different drying procedures (freeze-drying included) caused errors on the determination of Se by fluorescence spectrometry and of Mo, Cr, Co, Mn and Cu by atomic absorption spectrometry; thus, they designed a new drying apparatus which included a device for trapping evaporated substances [58]. Uchino *et al.* evaluated the stability of some elements (Fe, Zn, Cu, Co, Hg, Pb, As and Se) during lyophilization of rat liver using atomic absorption spectrometry after wet digestion of the sample matrix. Iron was confirmed to be extremely stable to lyophilization and was used as a reference element. The stabilities of the other elements were determined from the differences between the mean values of the element to Fe ratios for the wet and lyophilized samples. At a probability level of 0·05, only some Pb was found to be lost during lyophilization. Even so, 92% of the total amount of Pb was retained in the procedure. In another freezing-drying approach, the ratio of the freezing damage in the tissue samples to the formation of ice crystals and intracellular K/Na was measured [60]. A substantially decreased ratio was obtained. Contributions supporting the behaviour of Ca, Mg, and P in intracellular and extracellular electrolytes [61]; Na, K and Cl in intracellular electrolytes [62] and in biological soft tissues [63]; the P/K ratio in nuclei of bullfrog myocard cells [64]; Cd and Pb in seafood [65]; Zn, Co, Mn, Ru and Ce [66], Fe, Co, Cs, Se and Sr [57, 58] in various biological samples subjected to lyophilization have also been reported.

Gawlik *et al.* [69] used neutron activation analysis and biological liquid samples held in thin-walled quartz ampoules to irradiate and measure them directly without pretreatment. Losses of iron, zinc and selenium during pretreatment procedures (freeze-drying and oven-drying at 30, 60 and 90 °C) for the analysis of plasma, erythrocytes and liver were reported (they were less than 12% for all three elements).

A quality programme of analyses for toxic metals in urine was implemented in the Nordic countries between 1978

and 1987. In connection with this programme, the advantages and disadvantages of lyophilized compared to natural urine specimens as control materials were investigated in three similar studies. Three parallel lyophilized and natural specimens were distributed to 12 participating laboratories. Two of the three specimen pools were spiked with known amounts of As, Cd, Cr, Hg, Ni and Pb standard solutions. The results revealed no clear differences in the mean concentrations, coefficients of variation or mean recoveries for the various metals between the two control materials used with the various types of analytes. However, wide random variations were observed, emphasizing the analytical difficulty of these analyses and the need for routine quality control [70].

Trace-element sensitivities in proton-induced X-ray emission (PIXE) analysis were evaluated in serum samples prepared by freeze-drying and low-temperature ashing techniques. The latter target-preparation method yielded better detection limits for all elements. However, the gain in sensitivity, which is 60% at the most for low-Z elements, was not considered large enough to warrant the use of dry-ashing procedures in PIXE batch-analytical situations as is usually the case with biomedical work [71].

Proton and α-induced X-rays (determination of Fe, Cu, Zn and Br [72]) and SDS polyacrylamide gel electrophoresis (determination of sulphydryl and disulphide contents of soybean 11S globulin [73]) have also been used to monitor the effect of this type of pretreatment.

The use of freeze-drying in agricultural and food analysis has led to the solution of a variety of problems encountered in the storage and preconcentration of these kinds of samples.

Freeze-drying, freeze-substitution, and cryomicrotomy are most commonly used to examine the cellular location of labelled, diffusible compounds in plant tissues. However, it is not practical to process many samples at one time for microautoradiography if either cryomicrotomy or freeze-substitution are being used. With cryomicrotomy, sectioning and manipulating thin, frozen sections is difficult. Freeze-substitution has other disadvantages, namely very cold anhydrous conditions must be maintained during substitution and samples must be embedded immediately after it. In contrast, it is possible to freeze-dry many samples in a single experiment and store them under anhydrous conditions at room temperature for indefinite periods before embedding. Freeze-dried tissue is usually embedded by infiltrating with a nonpolar solvent, such as xylene or diethyl ether, and then passing the tissue through a graded solvent-resin series into epoxy resin. Vogelmann and Dickson [74] developed a modified pressure infiltration technique for the preparation of microautoradiographs of $^{14}C$-labelled, water-soluble compounds in plant tissue. Samples from cottonwood labelled with $^{14}C$ were excised, quick frozen in liquid $N_2$, freeze-dried at $-50\,°C$ and pressure-infiltrated with epoxy resin without intermediate solvents or prolonged incubation times. The technique facilitates the mass processing of samples for microautoradiography, provides good cellular retention of labelled water-soluble compounds, and is highly reproducible.

The sample preparation procedure for electron microprobe analysis in the study of soil-root interface [75], or the electron microscopic investigations by using critical point drying in preparing soils samples [76], the extraction of acid soluble phosphates of plants during the fixation of material by liquid nitrogen [77], and the comparison of the use of thermal and freeze-drying processes for storing food products prior to the determination of metal traces [78] testify to the research interest in these processes.

The use of freeze-drying on *aqueous samples* is usually aimed at preconcentration. The evaluation of lyophilization for preconcentration of natural water samples, prior to neutron activation analysis, showed that no consistent retention value was obtained for those elements (Hg and I) that were lost substantially during freeze-drying. Bromine, added as a bromide salt, was lost from acidified solutions, but quantitatively retained in neutral solutions. The results obtained with the three types of water assayed (filtered river water, tap-water, and deionized water) were indistinguishable. A critical factor in obtaining good retention yields, particularly for volatile elements, was to keep the sample frozen solid during the lyophilization process. Loss of vacuum is the primary reason for the sample melting, except in the case of saline samples [79]. Losses of Sb (as potassium antimony tartrate) and As (as dimethylarsenic acid or as sodium arsenate) and bromine (as bromide ion) during lyophilization of acidified and neutral aqueous synthetic and environmental samples ranged from zero to 60% for Sb and As, while losses of bromine were constant (at 91%) in acidic solutions. The variable losses of As and Sb were due solely to the presence and partial decomposition of the dimethylarsenic acid. Electrochemical oxidation of $Br^-$ to $Br_2$ was responsible for the high losses of bromine. In addition, losses of mercury (as dimethylmercuric chloride) were 100% in both acidic and neutral aqueous synthetic samples during lyophilization [80–85]. On the other hand, lyophilization has proved to be as effective as solvent extraction or chelating ion exchange for the concentration of Fe, Mn, Ni, Al, Cr, Cu and Cd from fresh-water samples [86].

Three quasi-independent analytical procedures for the determination of trace elements in rain-water were developed and compared by Stössel and Prange [87]. They are based on total reflection X-ray fluorescence (TXRF) and on three sample preparation techniques, namely direct analysis, pre-enrichment of the trace elements by freeze-drying and redissolution in dilute nitric acid, and a matrix removal and preconcentration procedure by metal chelation, chromatographic adsorption of the metal complexes and subsequent elution of the metal chelates prior to TXRF measurement. The elements determined were S, K, Ca, Tl, V, Cr, Mn, Fe, Co, Ni, Cu, Zn, As, Pb, Se, Rb, Sr, Mo, Cd, and Ba. For a measuring time of 1000 s, detection limits down to 5–20 ng/l were achieved for the heavy-metal traces. The limits were slightly higher for iron, nickel, copper, zinc, and lead because of fluctuation in the blank values. The procedures were tested on rain-water samples containing comparatively low trace-metal contents. Systematic investigations for the characterization of the analytical procedures with regard to blanks, detection limits, precision and accuracy were performed. The accuracy was checked by independent analyses of duplicate samples using differential pulse anodic stripping voltammetry. Of the three techniques presented, freeze-drying in conjunction with TXRF was the best because of its relatively simple and clean sample preparation procedure, which also resulted in low detection limits. However, in cases where the rainwater sample has high alkaline and alkaline-earth concentraions in the presence of extremely low trace-element content, it may be advisable to change over to the reverse-phase technique. The direct measurement should be applied in any case in order to get a first idea of the concentration range and the element composition of the sample, and finally to decide whether a pre-enrichment step is required [88].

Different studies on the microstructure and porosity of lyophilized aqueous solutions [89, 90], the determination of moisture [91, 92], of water activity [93], the control and determination of water during lyophilization [94–96] and other parameters have been performed [97–99].

*Organic compounds*

The influence of lyophilization on the features of organic compounds has been studied by number of authors, both in biological matrices and in aqueous solutions. The influence on plants and foods is considered separately because of their special features.

Freeze-drying, followed by infiltration with resin and polymerization by ultra-violet light at low temperatures and under constant vacuum, is an alternative tissue preparation technique for microprobe analysis. Embedding can be accomplished by a non-polar low-temperature resin which allows infiltration and polymerization at temperatures down to −50 °C. Sections of low temperature embedded material can be cut dry at −60 °C or at room temperature. Sectioning at low temperatures is an alternative for preparations that are difficult to cut at room temperature. The morphological preservation is adequate for the identification of structures such as mitochondria, lysosomes and different types of endoplasmic reticulum in liver cells. Thus, significant differences in the elemental composition can be detected between freeze-dried or freeze-substituted tissue prior to embedding. Freeze-drying is less time-consuming. By avoiding contact with organic solvents, the risks of ion losses and redistribution are diminished. In contrast to freeze-dried thin cryosections, low-temperature embedded material can be sectioned for light microscopy and areas of interest chosen for further thin and heterogeneous cell populations. The initial preparative step – the cryofixation – determines to a high degree the morphological preservation of freeze-dried and embedded tissue [100–102]. By using freeze-dried sections before microscopic observation one takes the risk of rehydration with its accompanying typical artefacts, which are prevented when freeze-dried sections are exposed to vapour fixatives (for example formaldehyde or osmium tetroxide). Dry vapour fixation enables the safe preserves of samples for microanalysis and offers the possibility of conducting immunocytochemical reactions on serial sections. This has been illustrated by Frederic *et al.* [103] by an immunocytochemical and microanalytical study on rat pancreas.

A recent study of phospholipids by $^{31}P$ nuclear magnetic resonance (NMR) after lyophilization or acetone desiccation of tissues showed specimen preservation in acetone to be a useful method for preserving tissue phospholipids for subsequent $^{31}P$ NMR profile analysis, and freeze-drying to be inadequate. Lipid extraction following a tissue acid extraction is also of little, or no, value in the determination of tissue phospholipid profiles [104]. Other studies on phospholipids [105], steroids [106, 107], lipids [108, 109] and fatty acids [110] yielded contradictory results in relation to the behaviour of these compounds in the lyophilization process, as well as in the study by Fujita *et al.* on the behaviour of protein, phospholipids and enzymes [111], and other studies on enzymes [112–116], coenzymes [117], ATP [118], androgens [119] and sex hormones [120, 121].

Cheng Meng *et al.* [122] developed a fast, convenient and inexpensive method for concentrating biogenic amines and their metabolites from biological samples for analysis by HPLC-EC. Recovery of standard monoamines and metabolites from artificial cerebrospinal fluid solution following lyophilization in the presence of glutathione and EGTA was higher than 89%; the coefficient of variation was 0·6–3·7%, depending on the specific concentrated amine or metabolite. Lyophilization as a one-step procedure was considered for concentrating biogenic amines and metabolites from biological fluids containing low concentrations of protein and other interfering substances. When concentrating compounds from plasma, which contains large quantities of protein and other electrochemically active materials, it is necessary to add an extraction step, such as alumina extraction. Recovery of endogenous catecholamines from plasma following the combined alumina extraction-lyophilization procedure was 81 ± 1%. On the other hand, Bartelik and Mikolajczyk [123] found lyophilization of human serum to cause a significant decrease in $\alpha_1$-lipoprotein and a smaller decrease in the β-lipoprotein and transferrin concentrations. Changes in the concentrations of other protein were insignificant [123]. Other studies on amino acids and proteins confirmed lyophilization as an excellent approach to storing and preconcentrating samples [124–129], although others detract from their use [130–132].

One method for detection of drugs in urine is based on lyophilization and liquid-liquid extraction. Urine samples are acidified with acetic acid and then freeze-dried. The residues are then extracted and subjected to thin-layer chromatography. The method affords a much higher recovery of all types of compounds than standard extraction procedures [133].

Much research which is difficult to systematize because of heterogeneity of the organic samples (preconcentration methods for electrophoresis [134], microdetermination of water in various organs [135], analysis of a 30-year-old bottle of lyophilized plasma [136], protein synthesis initiation [137], etc. [135–157]) yielded contradictory results in relation to the behaviour of lyophilized samples.

*Aqueous and organic solvent matrices*

Detailed studies on the application of freeze-drying to the preconcentration of organothiophosphorus pesticides in waters were performed by Bargnoux *et al.* [160, 161] in order to cut losses of these compounds from natural (especially bicarbonated) water. Such losses were probably due to the partial hydrolysis of pesticides resulting from the high alkalization of bicarbonated solutions during freeze-drying. The controlled acidification [158], addition of appropriate excipients or chemical treatment [159] to the samples prevents degradation, thereby ensuring the satisfactory results with compounds such as parathion and malathion [160, 161].

A comparative study of the removal of yellow organic matter from aliquots of the same fresh water sample by four common techniques, such as lyophilization, ion-exchange, ultrafiltration and organic solvent extraction, showed ultrafiltration to be superior for quantitation of the dissolved yellow organic fraction of natural waters. The lyophilization technique, which should also have recovered all the yellow organics, has the liability of requiring significant manipulation of the sample prior to freezing. Incomplete removal of the non-yellow organics and inorganics probably contributed to the low yield of the same fractions obtained by this technique. The ion-exchange technique was inefficient and hence unsuitable for quantitative work. However, if large quantities of yellow organics are required for various chemical analyses, ion-exchange has the advantage of enabling one to process large quantities of water conveniently and rapidly [162].

Studies on the quantitative freeze-drying of aqueous solutions of some metabolically important aliphatic acids prior to gas-liquid chromatographic analysis showed the overall losses observed to be due to volatilization during the freeze-drying process. The losses were related to the variation of the vapour or sublimation pressures of the acids with temperature, and also related to their latent heats of vaporization or sublimation. Reliable data on the latent heats of the acids of interest are seldom available, so a method for their estimation based on group contributions was developed. Thermochemical data derived from the use of this method were used in conjunction with reliable experimental data to determine the optimum freeze-drying conditions for the complete quantitative recovery of all but the most volatile of the acids studied, with a minimum of preliminary experimental work [163, 164].

The irregular behaviour of different organic compounds in water (nitrosamines [165], cobalamines [166], alcohols and ketones [167], carbohydrates [168], peptides [169], dibenzodioxins [170, 171], non-specific organic matter in river water [172, 173], organic pollutants [174, 175] and sludge [176], and other special studies [177–179]) allowed no final conclusions to be established.

The hydration of cytidine-5′-phosphoric acid was accomplished by freeze-drying Fourier Transform Infra-red Attenuated Total Reflection Spectroscopy [180]. The infra-red ATR spectrum of this compound was studied as a function of the relative humidity. The hydration pattern was shown to depend strongly on the initial water coverage of the crystal surface. Complete surface deuteration was accomplished. The involvement of most of the molecular subgroups of the analyte in the surface hydration was shown.

*Plants and food matrices*

Although lyophilization seems to be best procedure for storing and preconcentrating plant material and food after analysis, authors do not completely agree on the results. A study on the long-term preservation of eggs and tissue homogenates for the determination of organochlorine compounds, using freezing and freeze-drying processes, was performed by Norstrom and Won [181]. Storage of wet egg homogenates at temperatures from −18 to −28 °C was more suitable for long-term preservation than was freeze-drying. Changes in residue levels of heptachlor epoxide, oxychlordane, dieldrin, hexachlorobenzene, p,p′-DDE, mirex, and PCBs (polychlorinated biphenyls) were not significant over a three-year period in fresh herring-gull egg homogenates stored at −18 to −28 °C. Compounds with gas chromatographic retention 10 times shorter than hexachlorobenzene vaporized during freeze-drying at a rate proportional to their volatility.

Evaporative losses of components with vapour pressures less than hexachlorobenzene did not occur in naturally contaminated herring-gull eggs after storage at room temperature for up to one year. Higher losses of all compounds (up to 25% for p,p′-DDE) occurred in freeze-dried whole-body herring-gull homogenates. Easily dehydrochlorinated compounds were rapidly degraded in freeze-dried chicken egg homogenate at room temperature: the half life of p,p′-DDE and p,p′-DDD was about 20 days, and that of α- and γ-hexachlorocyclohexane was much shorter than 16 days. About one-third of oxychlordane in herring-gull eggs was lost in one year under these conditions, but none was lost after freeze-drying when the homogenate was stored at −18 to −28 °C.

The effect of lyophilization on high amylopectin starch was determined on samples of starch dispersed in water with or without solubilized sucrose. The X-ray diffraction data showed this treatment to disrupt the crystalline structures of amylopectin and sucrose, exposing additional sites for water sorption. This was also shown by the sorption isotherms of the starch alone. The Hylon 7-sucrose freeze-dried samples had a greater effect on sorption than the corresponding freeze-dried mixture Amioca did. Organic probe analysis of the starch and starch-sucrose mixtures indicated that high amylose starches were more reactive with polar organic probes than high amylopectin starches when freeze-dried. Untreated high amylopectin starches were more reactive with probes, which is consistent with the water sorption data for more extensively branched polymers [182]. In this respect, van Sumere *et al.* [183] concluded that the freeze-drying of biological material, which is to be quantitatively analysed (micro-amount level) for compounds of low or intermediate molecular weight, should either be omitted or handled under strict control. This is because such compounds as amino acids, sugars, flavonids, glycosides, coenzymes, peptides, etc., might be removed from concentrates and/or the ground biological material by the high vacuum [183].

Other studies on lyophilization of amino acids [184], milk [185], flour protein [186] and volatile plant exudates [187] showed more or less significant losses of analytes to occur in the lyophilization step. Nevertheless, other authors such as Haslemore *et al.* [188], who used four different methods to dry various materials, and then determined their content in nitrogen-soluble sugar and starch, concluded that recoveries of sugars and starch were best from freeze-drying; rapid heating (100 °C/1 h) followed by slow heat-drying (50 °C/23 h), or vacuum-drying at 40 °C were only marginally inferior for the recovery of starch and sugars, respectively. Substantial losses for both sugars and starch resulted from heat-drying at 95 °C. Total nitrogen recovery was highest in heat-dried and lowest in freeze-dried tissue. The tissues of some species held for 4 h under warm conditions before drying had significantly different soluble sugar and starch concentrations compared with tissues dried immediately after harvest [188].

The carotene content of fresh forages and silages analysed by two methods, including freeze-drying and grinding of the sample and chromatographing only one aliquot after extraction, was reported by Gillingham [189]. Data on orchard grass, fescue, clovergrass mixtures, and silage samples showed the method thus developed to be accurate and reproducible. The average variation was ±8 mg/g, and duplicate determinations yielded 16 identical values out of 34.

The determination of free amino acids and amino nitrogen in breweries showed lyophilization to be the optimal procedure – it resulted in minimal amino acid losses and the pressure rise on an analyser column did not exceed the required level [190]. Complete recovery of neutral sugars was achieved by Keeling and James [191]. Other authors' reports have indicated special precautions to be taken in order to achieve good results after freeze-drying [192, 193].

The retention of volatile components in liquid food during low-temperature drying and flux equations [194] and a diffusion model [195] have been reported. Experiments with ternary systems of water, sucrose and ethylacetate confirmed some of the predictions of this model. Chemical-technological aspects for retention [196] and concentration [197, 198] of aromatic substances were successfully developed in several types of apples [199–202], coffee [203], and strawberries, onions and 'sauerkraut' [204].

The method used for drying green tobacco-leaf samples for analysis affects the components of the leaf. The ideal procedure stops metabolic processes quickly after sampling. A comparison of the effects of drying the frozen tissue by heat (71 °C for 12 h) or by freeze-drying showed that for the components investigated (total nitrogen, nitrate, insoluble and soluble nitrogen, α-amino nitrogen, nicotine, reducing sugars, acids and pH) heat-drying to be practical for large bulk samples, but freeze-drying to be more efficient when metabolic processes are characterized [205].

Lyophilization, used in concentrating free fatty acids sodium salts in cheese, previously extracted with ether, substantially improves the recovery of volatile fatty acids in general and of acetic acid in particular [206].

On the other hand, the state of water in frozen liquid foods, which is supposed to influence the performance of freeze-drying, was studied by Kumagai *et al.* [207]. The amount of unfreezable water in milk was found to be constant, irrespective of the initial water content. As expected from the phase equilibrium, the water content of the non-ice part of the amorphous solution was constant among the samples with different initial contents. The volume fraction of ice crystals, which should be known in the analysis of the freeze-drying rate, was smaller at the low initial water content than that usually arrived at under the assumption that the water was all freezable.

*Matrices and the reasons for purpose freeze-drying samples*

The determination of residual moisture in dry biological preparations (plasma proteins [208], immunoglobulin G (IgG) [209]); the mutagenicity testing of drinking-water using freeze-drying extracts [210], the parametric analysis of self-freezing in an initially wet porous medium [211], and methodological studies of desiccation of composite restorations [212] and on the effect of convective heat transfer of the sublimation of a frozen semi-infinite porous medium [213, 214] are other aspects of research on freeze-drying in addition to the use of differential thermal analysis [215], freeze-dry methods for coating capillary columns [216], and lyophilized indicators [217].

*Pharmaceutical compounds*

Lyophilization is a common procedure in the pharmaceutical industry, but the coupling of this step with analytical studies of pharmaceutical preparations is not as common. Thus, only a few contributions to the titration of antihistaminic compounds in organic-aqueous emulsions after removal of water by lyopholization [218], studies on thermal analysis [219, 220], on microencapsulates [221] and on impurities [222] of freeze-dried pharmaceutical compounds have been reported in the literature, with no appropriate discussion of the advantages and disadvantages of the lyophilization step.

### Apparatus

In addition to commercially available lyophilizers, whose size makes them unsuitable for analytical purposes, other, smaller devices have appeared in the market in the last few years to meet specific needs. They were preceded by a series of customized lyophilizers designed for different analytical purposes.

A freeze-dry apparatus was constructed to remove free water from environmental samples for tritium determination. The apparatus is self-contained, it can process eight samples simultaneously and it uses a refrigeration system to avoid replenishment of the cold bath. Large samples (200–300 g) can be dried in three days [223].

Nakaguchi *et al.* designed a new drying apparatus equipped with a device for trapping evaporated substances which was used for pretreatment of biological samples prior to the determination of trace elements [58].

Some other auxiliary devices for lyophilizers have been designed for different purposes. Thus, a tray apparatus with several wells was designed for lyophilizing or freezing-drying biological substances, especially those as anticoagulants in blood gas analysis. The tray apparatus is suitable for producing a 'pledget' or single-unit dosage of predetermined USP values of freeze-dried biological material that can be stored for long periods of time in a sterile syringe to be used as blood anticoagulant. The tray has an upper plate laminate (covered by an insulator during freezing) with rows of wells for holding a biological solution of a preset concentration and a lower laminate, in the form of a sheet of conducting material, for establishing a temperature gradient across the height of the tray to assist in freezing the tray contents as the stackable trays sit on the cooling or heating shelves of the freeze-drier [224].

An instrument performing simultaneous $D_2$ (ratio of resistivity of ice to that of the resistivity of a product for a given temperature) and DTA techniques was described for use in control of freeze-drying processes. Its main components are a test chamber, a cooling and heating unit, a digital computer system and a printer. It was used to evaluate the thermal features of a 20% sucrose solution [225].

The preparation of uniform membrane filter standards for calibration of X-ray fluorescence spectrometers was described by Baum *et al.* [54]. The device used was an array of 37 capillary tubes of equal and known volumes mounted on an Al disk. When dipped in the standard solution, the capillaries were filled by capillary action. The device was then placed upon the membrane filter and the capillaries were drained simultaneously, wetting the entire filter. The filter standards were then freeze-dried. Standards were prepared for Cu, Zn, Ni, Pb and Mn and the deviation was less than $\pm 1\%$.

Trivedi *et al.* [226] constructed and described a rigid or semi-rigid disposable reaction container for measuring absorbance changes. A windowed reaction chamber was adapted for transmission of light and an auxiliary chamber was separated from the reaction chamber except for a small opening allowing the reagent to be forced through to trigger a reaction in the reaction chamber. The container may be used in an automated assay. It is also possible to store components of a lyophilized reagent in the compartments, store the container, then use it later for analysis (for example for the determination of lactate dehydrogenase in blood).

### Reviews

An earlier attempt at critically reviewing and comparing concentration processes for liquid foods was made in 1970 by Thijssen[227], but with very little information on the subject. An overview of freeze-drying in food technology was aimed at showing the importance of this step and its future prospects [228]. A review by Smith, on the other hand, was devoted to the influence of drying and storage conditions on non structural carbohydrates of herbage tissue [229], while its possibilities and applications in analytical chemistry were considered by Alonso Fernández in 1975 [230], as was its use prior to a gas chromatographic separation [231]. A review, with 21 references, appeared in 1977 on a very specific aspect of this process before X-ray microanalysis of diffusible elements [232]. The techniques for isolation and concentration of organic compounds in raw and treated waters reviewed by Wilson [233] also included lyophilization. The joint use of NMR and freeze-drying and of neutron activation analysis and freeze-drying [235] have also been considered. A more recent review by Nagy [236] rationalized the selection of the freeze-fracture freeze-drying (FFFD) method of biological bulk specimen preparation as well as the theoretical and practical problems of this method. The problems involved in specimen preparation, beam penetration and quantitative analysis of FFFD specimens were also dealt with.

### Conclusions

The general usefulness of freeze-drying in the different analytical fields has been shown through the discussions on the many applications described above.

Nevertheless, it is impossible to accurately establish the advantages and disadvantages of this process, and in which cases it is more suitable than other processes (such as freezing, extraction, etc.). The contradictory opinions of authors working in this field hinders the establishment of general guidelines. Such contradictions arise from three main reasons, namely:

(*a*) differences in lyophilizer designs (different powers involve different process times, while different volumes result in different freeze-drying behaviours).

(*b*) differences in the matrix features of similar samples used for the determination of the same analyte (for example blood serum, tissues from healthy and sick individuals, different foods, etc.).

(*c*) differences in working conditions.

Therefore, a very systematic and comprehensive study is still needed to finally delimit the applicability of freeze-drying to sample pretreatment (storage and preconcentration). This clarification will undoubtedly result in a wider use of this technique as a means of automatizing the preliminary steps of the analytical process.

### Acknowledgement

The authors would like to thank ECC for financial support (Contract No. CBM/ST/88–99).

### References

1. Foreman, J. K. and Stockwell, P. B., *Automatic Chemical Analysis and Topics in Automatic Chemical Analysis* (Ellis Horwood, Chichester, 1975 and 1979).
2. Manka, D. P. (Ed.), *Automates Stream Analysis for Process Control*, Vols I and II (Academic Press, London, 1982 and 1984).
3. Nauman, E. B. and Buffham, B. A., *Mixing in Continuous Flow Systems* (Wiley Interscience, New York, 1983).
4. Valcárcel, M. and Luque de Castro, M. D., *Automatic Methods of Analysis* (Elsevier, Amsterdam, 1988).
5. Bergamin, H. F., Krug, F. J., Zagatto, E. A. G., Arruda, E. C. and Coutinho, C. A., *Analytica Chimica Acta*, **190** (1986), 177.
6. Bergamin, H. F., Krug, F. J., Reis, B. F., Nobrega, J. A. Mesquita, M. and Souza, I. G., *Analytica Chimica Acta*, **214** (1988), 397.
7. Chen, D., Lázaro, F., Luque de Castro, M. D. and Valcárcel, M., *Analytica Chimica Acta*, **226** (1989), 221.
8. Lázaro, F., Luque de Castro, M. D., *Analytica Chimica Acta*, (in press).
9. Valcárcel, M. and Luque de Castro, M. D., *Journal of Chromatography*, **393** (1987), 3.
10. Valcárcel, M. and Luque de Castro, M. D., *Continuous Non-Chromatographic Separation Techniques* (Royal Society of Chemistry, 1990).
11. Lambert, P. H., *Journal of Chemical Laboratory Investigation*, **1** (1978), 1.
12. Greenquist, A. C., U.S. US4,461,829 (CI. 435–7; G01N33/54), 1984.
13. Jpn. Kokai Tokkyo Kobo JP 58 10.656 (83 10.656) (CI. G01N33/54), (1983).
14. Fonta, Isakondo, Koichi, Kobayashi, Takashi, Jpn. Kokai Tokkyo Kobo JP 60 188,067 (85, 188,067) (CI. C12N9/96), (1985).
15. Hagi, N., Inoue, M., Toyoda, K. and Tanaka, M., Jpn. Kokai Tokkyo Kobo JP 62, 151,758 (87,151,758) (CI. G01N33/543), (1987).
16. Hagi, N., Inoue, M. Toyoda, K. and Tanaka, M., Jpn. Kokai Tokkyo Kobo JP 62, 148,857 (CI. G01N33/543), (1987).
17. Nakagawa, N., Oyama, K. and Watanabe, S., Jpn. Kokai Tokkyo Kobo JP 62 42,061 (87, 42,061) (CI. G01N33/543), (1986).
18. Fujita, H., Kudo, K. and Unoki, M., Jpn. Kokai Tokkyo JP 62 209,362 (87,209,362) (CI. G01N33/543), (1987).
19. Bodner, A. and Ting, R. C. Y., Eur. Pat. Appl. EP 136,798 (CI. G01N33/569), (1985).
20. Kondo, K. Iwasa, S. and Yoshida, I. Eur. Pat. Appl. EP 49,896 (CI. G01N33/76), 1982.
21. Buergisser, E., Ger. Offen. DE 3,638,054 (CI. G01N33/531), (1988).
22. Reinhartz, A., Brit. UK Pat. Appl. GB 2,181,543 (CI. C12Q1/00), (1987).
23. Rouf, M. A. and Farrington, K. J., *Appl. Radiat. Isot.*, **38** (1987), 992.
24. Hagi, N. and Mitoma, N., Jpn. Kokai Tokkyo Kobo JP 63,111,467 (88,111,467) (CI. G01N33/543), (1988).
25. Sergeeva, M. G., Kurochkin, I. N., Skiyankina, O. A., Zaitaev, S. V. and Varfolomeev, S. D., *Neirokhimiya*, **5** (1986), 11.
26. Nikiforova, T. V., Kuznetsova, N. N. and Vavilova, L. M., *Laboratornoe Delo*, **3** (1988), 28.
27. Borghi, V. C. and Wajchenberg, B. L., *Arquivos Brasileiros de Endocrinologia e Metabologia*, **27** (1983), 86.
28. McCarthy, D. A., Field, M., Mumford, P., Moore, S. R. Holborrow, E. J. and Maini, R. N., *Journal of Immunological Methods*, **82** (1985), 155.
29. Kauffman, R. H., van Es, L. A. and Gaha, M. R., *Journal of Immunological Methods*, **31** (1979), 11.
30. Spadaro, A. M., McLoughlin, T. J. and Engler, Ph. V., Eur. Pat. Appl. EP 193,208 (CI. G01N33/546), (1986).
31. Yokoyama, S., Sano, M., Hakozaki, K. and Ohayashi, A., Jpn. Kokai Tokkyo Kobo JP 62,171,594 (87,171,694) (CI. C12P19/36), (1987).
32. University of Santiago, Sapin, 428,746 (CI. G01N), (1976).
33. Roland, M. D. and Berry, E., *Tech. Bull. Regist. Med. Technol.*, **38** (1968), 304.
34. Clark, P. M. S., Whitehead, T. P. and Kricka, L. J., *Anal. Chem. Symp. Ser.*, **6** (1981), 109.
35. Altman, P. L. In Dittmer, D. S. (Ed.), *Blood and Other Body Fluids* (FASEB, Washington D.C., 1961).
36. Wieland, H. and Seidel, D., *Clinical Chemistry*, **28** (1982), 1335.
37. Brunzel, J., Sniderman, A., Albers, J., Kwiterovich, P. Jr and Apoproteins, B., *Atherosclerosis*, **4** (1989), 79.
38. Vergani, C., Trovato, N. and Dioguardi, N., *Clinica Chimica Acta*, **87** (1987), 127.
39. Whayne, T., Alaupovic, P., Curry, E., Lee, E., Anderson, P. and Schechter, E., *Atherosclerosis*, **39** (1981), 411.
40. Henderson, L. O., Kazlehurst, J. S., Taylor, L. and Hannon, W. H., *Clinical Biochemistry*, **21** (1988), 219.
41. Degtyar, V. G., Konyukhova, L. K., Mozzhechkov, V. T. and Panina, A. A., *Laboratornoe Delo*, **10** (1983), 28.
42. Lawson, N. S., Haven, M. D. G. T. and Moore, T. D., *American Journal of Clinical Pathology*, **68** (1977), 117.
43. Glick, J. H., Jr., *Clinical Chemistry*, **23** (1977), 781.
44. Delderfield, A. J., Sutherland, I. A. and Campbell, P. J., *Journal of Biological Standards*, **6** (1978), 331.
45. Rehner, H., Barti, K. and Potenhauser, R., Ger. Offen. De 3,324,626 (CI. G01N33/96), (1985).
46. Hill, J. L. and Winfrey, L. J., U.S. US 4,731,330 (CI. G01N31/00), (1988).
47. Keyser, J. W, Watkins, G. Ll. and Salaman, J. R., *Clinical Chemistry*, **22** (1976), 926.
48. Craft, N. E., Byrd, G. D. and Hilpert, L. R., *Analytical Chemistry*, **61** (1989), 540.
49. Bristow, A. F., Dunn, D. and Tarelli, E., *Journal of Biological Standards*, **16** (1988), 55.
50. Iyengar, V., Wolf, W. R. and Tanner, J., *Fresenius Z. Anal. Chem.*, **332** (1988), 549.
51. Kramer, G. N., Angelis, L. de, Pauwels, J., Ooghe, W. and Belliardo, J. J., *Fresenius Z. Anal. Chem.*, **332** (1988), 694.
52. Versieck, J., Vanballenberghe, L., de Kesel, A., Hoste, J., Wallaeys, B., Vandenhaute, J., Baeck, N., Steyaert, H., Byrne, A. R. and Willian Sunderman, F. Jr., *Analytical Chimica Acta*, **204** (1988), 63.
53. Wong, C. M., Report 1974, UCID-16659, 7 pp., (1974).
54. Baum, R. M., Willis, R. D., Walter, R. L., Gutknecht, W. F. and Stiles, A. R., *X-Ray Fluoresc. Anal. Environ. Samples*, **165** (1977), 73.
55. Fourte, H. O. and Peisach, M., *Radiochem., Radioanal. Lett.*, **26** (1976), 277.
56. Iyengar, G. V., Kasperek, K. and Feinendegen, L. E., *Analytica Chimica Acta*, **138** (1982), 355.
57. Xue, Z. L. and Wang, Y. X., *J. Radioanal. Nucl. Chem.*, **119** (1987), 425.
58. Nakaguchi, Y., Morita, M., Fujino, O., Aono, T. and Hiraki, K., *Kinki Daigaku Kenkyu Hokoku*, **22** (1986), 87.
59. Uchino, E., Jin, K., Tsuzuki, T. and Inoue, K., *Analyst*, **112** (1987), 291.
60. Hirche, H., Heinrichs, J., Schaefer, H. E. and Schramm, M., *Fresenius Z. Anal. Chem.*, **308** (1981), 224.
61. Zierold, K. and Schaefer, D., *Verh. Dtsch. Zool. Ges.*, **80** (1987), 111.

62. Ingram, F. D. and Ingram, M. J., Sci. Biol. Specimen. Prep. Microsc. Microanal., Proc. Pfefferkorn Con. 2nd (1984), 167, 74.
63. Doerge, A., Rick, R., Gehring, K. and Thurau, K., *Pfluegers Arch.*, **373** (1978), 85.
64. Meyer, R., Schmitz, M. and Zierold, K., *Scanning Electron Microscopy*, **1** (1985), 419.
65. Kasano, K., Kusaka, M. and Yamashita, K., Shin Daikyowa Sekiyu Kagaku K.K., Yokkaichi, Japan, **16** (1978), 443.
66. Huljev, D., Huljev, B. and Rajkovic-Huljev, Z., *Radiol. Lugosl.*, **22** (1988), 403.
67. Wang, Y., Qin, J., Ji, Q., Wu, S., Wang, X. and Zhang, Y., *Huanking Kexue*, **6** (1985), 30.
68. Pietra, R., Ubertalli, L., Monaktala, P. and Sabbioni, E., Comm. Eur. Communities EUR, (1984), EUR 9211.
69. Gawlik, D., Berthold, K., Chisela, F. and Bratter, P., *J. Radional. Nucl. Chem.*, **112** (1987), 309.
70. Valkonen, S., Jarvisalo, J. and Aitio, A., *Ann. Clim. Lab. Sci.*, **17** (1987), 345.
71. Lecomte, R., Landsberger, S. and Monaro, S., *Int. J. Appl. Radiat. Isot.*, **33** (1982), 121.
72. Barette, M., Lamoureux, G., Lebel, E., Lecomte, R., Paradis, P. and Monaro, S., *Nucl. Instrum. Methods*, **134** (1976), 189.
73. Hoshi, Y. and Yamauchi, F., *Agric. Biol. Chem.*, **47** (1983), 2435.
74. Volgelmann, T. C. and Dickson, R. E., *Plant Physiology*, **70** (1982), 606.
75. Shi, W., Xu, M. and Liu, Z., *Turang Xuebao*, **24** (1987), 286.
76. Tippkoetter, R., *Z. Pflanzenernaehr. Bodenkd*, **148** (1985), 92.
77. Sokolov, O. A., *Agrokhimiya*, **9** (1973), 133.
78. Tereshchenko, A. P., Pokrovskaya, E. I., Filatkina, L. A. and Livanskaya, O. G., *Izv. Vyssh. Uchebn. Zaved., Pishch. Tekhnol.*, **6** (1977), 146.
79. Schutz, D. F. and Tureklan, K. K., *Geochim. Cosmochim. Acta* **29** (1965), 259.
80. Carison, M. and Litman, R., *Radiochem. Radioanal. Lett.*, **35** (1978), 161.
81. Vizhenskii, V. A., Andrievskii, E. I., Molchanova, T. P., Panova, R. T., Trofimenko, S. V. and Byr'ko, V. M., *Yad. Fiz. Metody Anal. Kontrole Okruzh. Sredy*, **129** (1985), 34.
82. Harrison, S. H., LaFleur, P. D. and Zoller, W. H., *Analytical Chemistry*, **129** (1975), 1685.
83. Lieser, K. H., Calmano, W., Heuss, E. and Neitzert, V., *J. Radioanal. Chem.*, **37** (1977), 717.
84. Foldzinka, A. and Zmijewska, W., *Radiochem. Radioanal. Lett.*, **27** (1976), 225.
85. Qian, X. and Li, X., *Huanjing Huaxue*, **5** (1986), 60.
86. Hall, A. and Casimiro Godinho, M., *Analytica Chimica Acta*, **113** (1980), 369.
87. Stossel, R. P. and Prange, A., *Analytical Chemistry*, **57** (1985), 2880.
88. Fenkart, K., Eng, E. and Frey, U., *Fresenius Z. Anal. Chem.*, **293** (1978), 364.
89. Shinagawa, H. and Kawamura, Y., *J. Chem. Eng. Jpn.*, **12** (1979), 406.
90. Oleinikov, N. N., Shiyakhtin, O. A. and Tret'yakov, Y. D., *Therm. Anal.*, **2** (1985), 309.
91. Lebedev, D. P., Baisiev, Kh. and Andreev, E. F., *Teor. Osn. Khim. Tekhnol.*, **17** (1983). 259.
92. Kassai, L. and Ezer, E., *Gyogyszereszet*, **15** (1971), 225.
93. Blankov, B. I., Kaz'mina, Y. G. and Terent'eva, V. B., *Vaktsiny Syvorotki, Mosk. Nawch. Issled. Inst. Vaksin Syvorotov*, **5** (1965), 159.
94. Simatos, S., Fr. 1,463,174 (CI. B01d, F26b, G01n), (1966).
95. Koening, I., Knauth, H. D., Koopmann, C., Wagner, F. E. and Wagner, U., *Hyperfine Interact.*, **41** (1988), 811.
96. Tachimori, S., *J. Nucl. Sci. Technol.*, **13** (1976), 442.
97. Patel, M., *Trans. Indian Ceram. Soc.*, **41** (1982), 29.
98. Le Roy, A. and Roinel, N., *Electron. Microsc. Proc. Eur. Congr.*, **3** (1980), 161.
99. Wroblewski, J. and Wroblewski, R., *J. Microsc.*, **142** (1986), 351.
100. Zierold, K., X-ray microanalysis of frozen-hydrated specimens. *Scanning Electron Microsc.*, **2** (1983), 809.
101. Wroblewski, R., Wroblewski, J., Anniko, M. and Edstrom, L., *Scanning Electrom.*, **1** (1985), 447.
102. Burns, M. S., Chabala, J., Hallegot, P. and Levi-Setti, R., *Microbeam Anal.*, **23** (1988), 445.
103. Frederik, P. M., Bomans, P. H. H., Busing, W. M. and Odselius, R., *J. Phys. Colloq.*, **C2** (1984), 451.
104. Meneses, P., Para, P. F. and Glonck, T., *J. Lipid Res.*, **30** (1989), 458.
105. Boesterling, B. and Stier, A., *Biochim. Biophys. Acta*, **729** (1983), 258.
106. Das, R. E. G., Calam, D. H., Mitchell, F. L., Woofford, F. P., Cawood, M., Gaskell, S. J., Joyce, B., McMillan, M. and Wheeler, M. J., *Ann. Clin. Biochem.*, **20** (1983), 364.
107. Degtyar, V. G., Miloserdov, N. E. and Yu, V., U.S.S.R. SU 1,315,903 (CI. G01N33/50), (1987).
108. Oda, S., Nakatsuka, Ch. and Urata, T., *Rinsho Byori*, **33** (1985), 1165.
109. Mutti, Ch. M. L., Wu, T. W. and Lynn, S. Y., U.S. 4,127,502 (CI. 252–408, G01N33/16), (1978).
110. Jiménez, J. and Larsson, L., *J. Clin. Microbiol.*, **24** (1986), 844.
111. Fujita, S. C., Inoue, H., Yoshioka, T. and Hotta, Y., *Biochemical Journal*, **243** (1987), 97.
112. Brojer, B. and Moss, D. W., *Clinica Chimica Acta*, **35** (1971), 511.
113. Pienkos, Ph. T., Shah, V. K. and Brill, W. J., *Proc. Natl. Acad. Sci.*, **74** (1977), 5468.
114. Zhao, X. and Zhang, T., *Shandong Yike Daxue Xuebao*, **24** (1986), 42.
115. Stelzner, A., Holtz, H., Klein, U., Klein Y. M. and Schmidt, J., *Allerg. Immunol.*, **28** (1982), 28, 251.
116. Desjarlais, F., *Ann. Biochim. Clin. Que.*, **24** (1985), 136.
117. Zhao, Z., Hu, X. and Ross, C. W., *Plant Physiol.*, **84** (1987), 987.
118. Larionov, V. N., Mokhova, E. N., Priorova, N. I. and Tarakanova, A. N., *Biol. Nauki*, **2** (1988), 105.
119. de Larminat, M. A., Bruchovsky, N. and Rennie, P. S., *J. Steroid Biochem.*, **16** (1982), 811.
120. Attramadal, A., *Histochemie*, **19** (1969), 75.
121. Attramadal, A., *Histochemie*, **19** (1969), 110.
122. Cheng Meng, Q., Chen, Y. F. and Oparil, S., *Life Sci.*, **44** (1989), 1207.
123. Bartelik, S. and Mikolajczyk, S., *Probl. Tech. Med*, **11** (1980), 123.
124. McNeill, T. H. and Sladek, J. R. Jr, *Brain Res. Bull.*, **5** (1980), 599.
125. Moodie, I. M., *Laboratory Practice*, **29** (1980), 1074.
126. Jorgensen, A. O. and McGuffee, L. J., *J. Histochem. Cytochem.*, **35** (1987), 723.
127. Cenciarelly, M. *Diss. Abstr. Int.*, **47** (1986), 1346.
128. Knyazev, G. G., *Ser. Biol. Nauk*, **1** (1974), 101.
129. Tung, H. W. and Chiang, Ch. H., *Sheng Wu Hua Hsueh Yu Sheng Wu Wu Li Gsueh Pao*, **13** (1981), 117.
130. Guise, M. B., Gvrazdauskas, F. C. and Lineweaver, J. A., *Proc. Soc. Exp. Biol. Med.*, **167** (1981), 290.
131. Coulter, H. D., *J. Electron. Microsc. Tech.*, **4** (1986), 315.
132. Goodall, A. A. and Marshall, R. D., *Biochemical Journal*, **189**, (1980), 553.

133. Broich, J. R., Hoffman, D. B., Goldner, S. J., Andryauskas, S. and Umberger, J., *Journal of Chromatography*, **63** (1971), 309.
134. Yoshizawa, I., *Kensa to Gijutsu*, **9** (1981), 339.
135. Shibata, T., Inayama, S., Makamura, K., Nakasato, Y. and Hosoda, Y., *Igaku no Ayumi*, **113** (1980), 413.
136. Fu, F. P. and Myhre, B., *Transfusions*, **1** (1977), 73.
137. Ahmad, M. F., 1984, 85 pp.
138. Kalichava, G. S., *Soobshch Akad. Nauk Gruz*, **54** (1969), 213.
139. Sano, K., *Med. Technol.*, **8** (1980), 581.
140. Zierold, K., *Scanning Electron Microsc.*, **4** (1984), 1867.
141. DeLosch, J. R. and Holman, G. M., *Comp. Biochem. Physiol.*, **75A** (1983), 499.
142. Ianzini, F. and Rosi, A., *Ist. Super. Sanita, Lab. Radiaz.*, **82** (1982), 2.
143. Clayton, C. J., McNeill, T. H. and Sladek, J. R. Jr., *Cell Tissue Reserarch*, **220** (1981), 223.
144. Pool, T. B., Smith, N. K. R., Doyle, K. H. and Cameron, I. L., *Cytobios.*, **28** (1980), 17.
145. Baker, J. R. J. and Appleton, T. C., *Journal of Microscopy*, **108** 307.
146. Sjostrand, F. S., *J. Ultrastuct. Res.*, **53** (1975), 1.
147. Kobayashi, T. and Bakay, L., *Journal of Medicine*, **2** (1971), 35.
148. Hellman, B. and Lernmark, A., *J. Histochem. Cytochem.*, **19** (1971), 186.
149. Fleury, C., *Arch. Sci.*, **18** (1965), 588.
150. Baran, T., Rusu, V., Baciu, L., Mindreci, L. and Vasiliu, I., *Rev. Med. Chir.*, **90** (1986), 491.
151. Zhao, B., Duan, S., Zhang, J. and Xin, W., *Fenzi Kexue Yu Huaxue Yanjiu*, **4** (1984), 221.
152. Ingram, F. D. and Ingram, M. J., *J. Microsc. Biol. Cell*, **22** (1975), 193.
153. Wroblewski, R. and Wroblewski, J., *Histochemistry*, **81** (1984), 469.
154. Powell, R. J., Eur. Pat. Appl. EP 96,520 (CI. G01N33/54), (1983).
155. Young, J. W. and Christian, G. D., *Analytical Chemistry*, **45** (1973), 1296.
156. Toribara, T. Y. and Di Stefano, V., *Analytical Chemistry*, **26** (1954), 1519.
157. Mosulishvili, L. M., Kharadze, N. E, Belokobyl'skii, A. I. and Gudushauri, A., *Soobshch. Akad. Nauk Gruz.*, **98** (1980), 685.
158. Bargnoux, H., Pepin, D., Chabard, J. L., Vedrine, F., Petit, J. and Berger, J. A., *Analusis*, **6** (1978), 107.
159. Pepin, D. and Bargnoux, H., *J. Francais d'Hydrollgie*, **9** (1978), 125.
160. Bargnoux, H., Pepin, D., Chabard, J. L., Vedrine, F., Petit, J. and Berger, J. A., *Analusis*, **4** (1977), 170.
161. Bargnoux, H., Pepin, D., Chabard, J. L., Vedrine, F., Petit, J. and Berger, J. A., *Analusis*, **8** (1980), 117.
162. Milanovich, F. P., Ireland, R. R. and Wilson, D. W., *Environ. Lett.*, **8** (1975), 337.
163. Chalmers, R. A. and Watts, R. W. E., *Analyst*, **97** (1972), 224.
164. Jart, A., *Acta Polytech. Scand. Chem. Incl.Metall. Ser.*, **121** (1974), 116.
165. Du Plessis, L. S. and Nunn, J. R., Anal. Dorm. Proc. Work. Conf., **55** (1951), 63.
166. Beck, R. A. and Brink, J. J., *Environ. Sci. Technol.*, **10** (1976), 173.
167. Sadowski, L. H. and Harris, J. C., ADL-82480-19, EPA-600/2-84-012, (1984).
168. To, E. C. and Flink, J. M., *J. Food Technol.*, **13** (1978), 551.
169. Margolis, S. A. and Longenbach, P. J., *Role Pept. Neuronal. Func.*, **49** (1980), 67.
170. Stieglitz, L., Zwick, G. and Roth, W., *Chemosphere*, **15** (1986), 1135.
171. Orcharova, G., Vitanov, T. and Angelova, V., *Izv. Durzh. Inst. Kontrol Lek. Sredstra*, **11** (1978), 49.
172. Sirotkina, I. S., Zagudaeva, N. S. and Varshal, G. M., *Gidrokhim. Mater.*, **53** (1972), 147.
173. Kunte, H., Pfeiffer, E. H., *Bull. Environ. Contam. Toxicol.*, **34** (1985), 650.
174. Urano, K., Yao, S. Ch. and Hayashi, K., *Bull. Chem. Soc. Jpn.*, **56** (1983), 1271.
175. Uno, M., Onji, Y., Nakahira, H. and Tanigawa, K., *Nara-ken Eisei Kenkyusho Nenpo*, **18** (1983), 73.
176. Bauer, I. and Thomanetz, E., *Gas-Wasserfach Wasser Abwasser*, **118** (1977), 538.
177. Ross, W. D., Hillan, W. J., Wininger, M. T., Gridley, J. A., Lee, L. F., Hare, R. J. and Sandhy, S. S., *Environ. Sci. Res.*, **22** (1981), 179.
178. Koszterszitz, G., Barnikol, W. K. R. and Schulz, G. V., *Makromol. Chem.*, **178** (1977), 1135.
179. Jennings, T. A., *J. Parenter. Drug. Assoc.*, **34** (1980), 62.
180. Bailescu, S. and Sandorfy, C., *Appl. Spectrosc.*, **40** (1980), 1236.
181. Norstrom, R. J. and Won, H. T., *J. Assoc. Anal. Chem.*, **68**, (1985), 129.
182. Castillo, P. J., Gilbert, S. G. and Daun, H., *J. Food Sci.*, **54** 1989, 162.
183. van Sumere, C., Geiger, H., Bral, D., Fockernier, G. Vande Casteele, K., Martens, M., Hanselaer, S. and Gevaert, L., *Anal. Biochem.*, (131 (1983), 530.
184. Bauer-Staeb, G. and Bouvard, F., *Lebensm. Wiss. Technol.*, **6** (1973), 219.
185. Gordin, S., Birk, Y. and Volcani, R., *J. Dairy Sci.*, **55** (1972), 1544.
186. Kasano, K., Kusaka, M. and Yamashita, K., *Shin Daikoyowa Sekiyu Kagaku*, **16** (1978), 385.
187. Skvortsov, S. S., *Mater. Vses. Simp. Fiziol. Biokhim. Osn. Form. Rast. Soobshcheste* (1965), 302.
188. Haslemore, R. M., Warrington, I. J. and Rougham, P. G., *N.Z.J. Agric. Res.*, **23** (1980), 355.
189. Gillingham, J. T., *J.A.O.A.C.*, **50** (1967), 828.
190. Basarova, G. and Cerna, I., *Kvasny Prum.*, **18** (1972), 78.
191. Keeling, P. L. and James, Ph., *J. Liq. Chromatogr.*, **9** (1986), 983.
192. Fisher, D.S . and Burns, J. C., *Agron. J.*, **79** (1987), 236.
193. van Sumere, C., Geiger, H., Bral, D., Fockernier, G., Vande Casteele, K., Martens, M., Hanselaer, R. and Gevaert, L., *Anal. Biochem.*, **131** (1982), 530.
194. Santosh, K. and King, C. J., *A.I.Ch.E.J.*, **18** (1972), 520.
195. King, C. J. and Chandrasekaran, S. K., *Prog. Refrig. Sci. Technol. Proc. Int. Congr. Refrig.*, **3** (1971), 649.
196. Pozderovic, A. and Lovric, T., Prehrambeno-Technol. Biotehnoloska Rev., **24** (1986), 101.
197. Drawert, F., Schreier, P., Bhiwapurkar, S. and Heindze, I., *Weuman Symp.*, (1981), 649.
198. Flink, J. M. and Labuza, T. P., *J. Food Sci.*, **37** (1972), 617.
199. Hawkes, J. and Flink, J. M., *J. Food Preserv.*, **2** (1978), 265.
200. Pereira, A. Jr and Borralho da Graca, J., *García Orta*, **14** (1964), 497.
201. Gorin, N. and Zonneveld, H., *Lebensm. Wiss. Technol.*, **10** (1977), 50.
202. Popovskii, V. G., Timofeeva, O. A. and Soboleva, I. M., *Tr. Mold. Nauch. Issied. Inst. Pishch. Prom.*, **8** (1968), 82.
203. Quijano Rico, M., Díaz, S. J. and Fritsch, G., *Colloq. Int. Chim. Cafes*, **6** (1973), 184.
204. Florin, U., *Ind. Obst. Gemueseverwert*, **54** (1969), 680.
205. Gous, P. J., Kroontje, W. and Terrill, T. R., *Tob. Sci.*, **14** (1970), 63.
206. Hote-Baudart, E., *Bull. Rech. Agron. Gembloux*, **3** (1968), 689.

207. Kumagai, H., Nakamura, K. and Fujiwhara, J., *Agric. Biol. Chem.*, **49** (1985), 3097.
208. Podol'skii, M. V., Epifanov, S. N. and Skur'yat, E. N., *Lab. Delo,* **10** (1975), 623.
209. Kluss, E. M. and Mester, T., *Aerztl. Lab.*, **23** (1977), 339.
210. Forster, R., *Soc. Appl. Bacteriol. Tech. Ser.*, **19** (1984), 375.
211. Fey, Y. C. and Boles, M. A., *Int. J. Heat Fluid Flow,* **9** (1988), 147.
212. Roulet, J. F. and Michellod, P. Y., *Schweiz. Monatsschr. Zahnmed.,* **94** (1984), 1049.
213. Fey, Y. C. and Boles, M. A., *Int. J. Heat Mass Transfer,* **30** (1987), 771.
214. Fey, Y. C. and Boles, M. A., *Int. J. Heat Mass Transfer,* **31** (1988), 1645.
215. Soler Peix, E., *Cienc. Ind. Farm.*, **6** (1974), 135.
216. Harrison, I. T., *Analytical Chemistry,* **47** (1975), 1211.
217. Alonso Fernández, J. R., Bermejo Martínez, F. and Sánchez, A. C., *Acta Cient. Compostelana,* **12** (1975), 1.
218. Viola, M. R., Alboini, A. M., Canestrini, C. and Donelli, G., *Boll. Chim. Farm.,* **110** (1971), 110, 349.
219. Phillips, A. J., Yarwood, R. J. and Collett, J. H., *Anal. Proc.,* **23** (1986), 394.
220. Soler Peix, E., *Cienc. Ind. Farm.,* **7** (1975), 131.
221. Yoon, M. A. and Yong, J. I., *Yakche Hakhoechi,* **17** (1987), 67.
222. Angeli, M. G., Mercandalli, G., Minoja, F., Tedeschi, S. and Cingotani, *Farmaco Ed. Prat.,* **35** (1980), 100.
223. Hayes, D. W., *Report* (1982), DP-1634, 17 pp.
224. Williams, C. D., US 4,521,719 (CI. 422-102; B01L3/00), (1985).
225. Jennings, T. A., *Med. Device Diagn. Ind.,* **2** (1980, 49.
226. Trivedi, R. C., Bungay III, H. R. and Cuccolo, P. J., US 4,040,786 (CI. 23-230R; G01N33/16), (1977).
227. Thijssen, H. A. C., *J. Food Technol.,* **5** (1970), 211.
228. Karel, M., *Proc. R. Soc. London,* **191** (1975), 21.
229. Smith, D., *J. Brit. Grassl. Soc.,* **28** (1973), 129.
230. Alonso Fernández, J. R., *Quim. Anal.,* **30** (1976), 339.
231. Alonso Fernández, J. R., *Acta Cient. Compostelana,* **20** (1983), 221.
232. Appleton, T. C., *Soc. Exp. Biol. Semin. Ser.,* **33** (1977), 247.
233. Wilson, I., *Methodol. Surv.,* **10** (1981), 76.
234. Suzuki, E. and Nagashima, N., *Keisoku Gijutsu,* **11** (1983), 50.
235. Qin, Y., *Hejishu,* **11** (1988), 1.
236. Nagy, I. Z., *Scanning Microsc.,* **2** (1988), 301.

